# Virtual reality gameplay classification illustrates the multidimensionality of visuospatial neglect

**DOI:** 10.1093/braincomms/fcae145

**Published:** 2024-05-03

**Authors:** David R Painter, Michael F Norwood, Chelsea H Marsh, Trevor Hine, Christie Woodman, Marilia Libera, Daniel Harvie, Kelly Dungey, Ben Chen, Julie Bernhardt, Leslie Gan, Susan Jones, Heidi Zeeman

**Affiliations:** The Hopkins Centre, Menzies Health Institute Queensland, Griffith University, Nathan, Queensland, 4111, Australia; The Hopkins Centre, Menzies Health Institute Queensland, Griffith University, Nathan, Queensland, 4111, Australia; The Hopkins Centre, Menzies Health Institute Queensland, Griffith University, Nathan, Queensland, 4111, Australia; School of Applied Psychology, Griffith University, Gold Coast, Queensland, 4215, Australia; The Hopkins Centre, Menzies Health Institute Queensland, Griffith University, Nathan, Queensland, 4111, Australia; School of Applied Psychology, Griffith University, Mount Gravatt, Queensland, 4215, Australia; Neurosciences Rehabilitation Unit, Gold Coast University Hospital, Gold Coast, Queensland, 4215, Australia; Psychology Department, Logan Hospital, Logan, Queensland, 4131, Australia; The Hopkins Centre, Menzies Health Institute Queensland, Griffith University, Nathan, Queensland, 4111, Australia; Allied Health and Human Performance, Innovation, Implementation and Clinical Translation in Health (IIMPACT in Health), University South Australia, Adelaide, 5001, South Australia, Australia; School of Applied Psychology, Griffith University, Mount Gravatt, Queensland, 4215, Australia; Allied Health and Rehabilitation, Emergency and Specialty Services, Gold Coast Health, Gold Coast, Queensland, 4215, Australia; Florey Institute of Neuroscience and Mental Health, Austin Campus, Heidelberg, 3084, Victoria, Australia; Rehabilitation Unit, Logan Hospital, Meadowbrook, Queensland, 4131, Australia; School of Applied Psychology, Griffith University, Mount Gravatt, Queensland, 4215, Australia; The Hopkins Centre, Menzies Health Institute Queensland, Griffith University, Nathan, Queensland, 4111, Australia

**Keywords:** brain injury, cognitive assessment, classification, immersive virtual reality, unilateral visuospatial neglect

## Abstract

Brain injuries can significantly impact mental processes and lead to hidden disabilities not easily detectable. Traditional methods for assessing these impacts are imprecise, leading to unreliable prevalence estimates and treatments with uncertain effectiveness. Immersive virtual reality has shown promise for assessment, but its use as a standalone tool is rare. Our research focused on developing and validating a standalone immersive virtual reality classification system for unilateral spatial neglect, a condition common following brain injury characterized by inattention to one side of space. Our study involved 51 brain injury inpatients and 30 controls, all engaging with ‘The Attention Atlas’, an immersive virtual reality game for testing visual search skills. Our classification system aimed to identify patients with neglect, ‘minor atypicality’ (indicative of inattention not consistent enough to be labelled as neglect) or non-neglect. This categorization was based on a simple mathematical definition, utilizing gameplay to describe spatial orientation (to the left or right side) and attentional challenge (indicative of search inefficiency). These metrics were benchmarked against a normative model to detect atypical visual search, which refers to gameplay beyond the usual bounds. The combination of neglected side, orientation and challenge factors was used to categorize neglect. We discovered a strong correlation between atypical visual search patterns and neglect risk factors, such as middle cerebral artery stroke, parietal injuries and existing neglect diagnoses (Poisson regression incidence rate ratio = 7.18, 95% confidence interval = 4.41–11.90). In our study, immersive virtual reality-identified neglect in one-fourth of the patients (*n* = 13, 25.5%), minor atypicality in 17.6% (*n* = 9) and non-neglect in the majority, 56.9% (*n* = 29). This contrasts with standard assessments, which detected neglect in 17.6% (*n* = 9) of cases and had no intermediate category. Our analysis determined six categories of neglect, the most common being left hemispace neglect with above-median orientation and challenge scores. Traditional assessments were not significantly more accurate (accuracy = 84.3%, *P* = 0.06) than a blanket assumption of non-neglect. Traditional assessments were also relatively insensitive in detecting immersive virtual reality-identified neglect (53.8%), particularly in less severe cases and those involving right-side inattention. Our findings underline the effectiveness of immersive virtual reality in revealing various dimensions of neglect, surpassing traditional methods in sensitivity and detail and operating independently from them. To integrate immersive virtual reality into real-world clinical settings, collaboration with healthcare professionals, patients and other stakeholders is crucial to ensure practical applicability and accessibility.

## Teaser

Painter *et al*. validated ‘The Attention Atlas’, an immersive virtual reality game for detecting and categorizing unilateral spatial neglect, a brain injury condition characterized by inattention to one side of space. The system shows greater sensitivity than traditional methods, showing the potential of immersive virtual reality for neglect assessment and treatment.

## Graphical Abstract

**Graphical Abstract:**
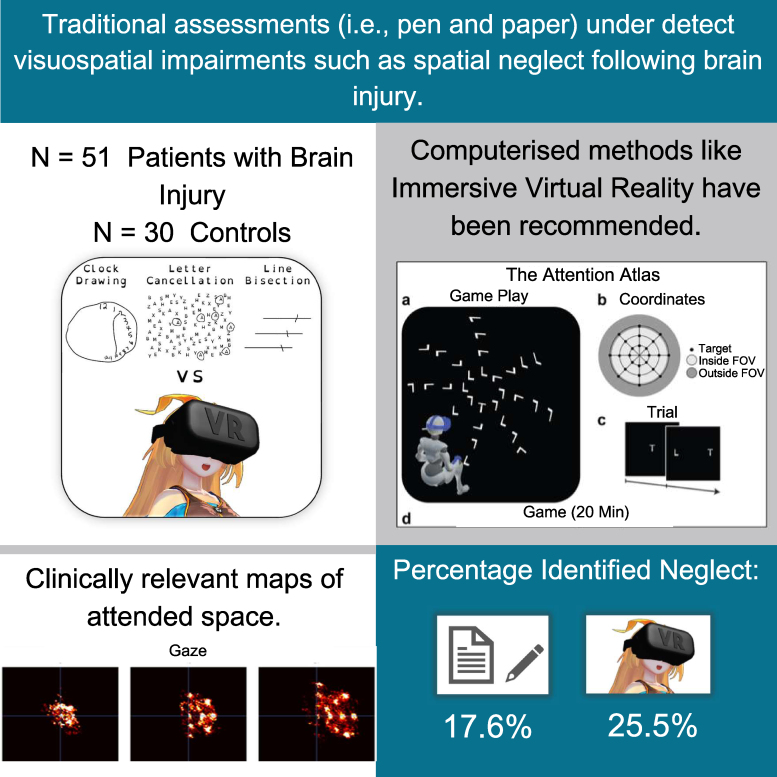

## Introduction

### Unilateral spatial neglect

Unilateral spatial neglect, which can render the contralesional hemispace inaccessible to visuospatial attention,^1-4^ is common following brain injury.^5,6^ A person with neglect may perceive half of their visual world as non-existent^7^ despite intact vision.^8^ Notable characteristics of neglect include (i) orientation towards the ipsilesional hemispace, manifesting in the head,^9,10^ limb^3,11^ and eye movements^12-14^; (ii) inattention to objects in the contralesional hemispace, manifesting in reduced detection and slowed responses^15-17^; and (3) limited attentional capacity^18^ manifesting with task demand,^19^ scene complexity^20^ and interstimulus competition^21,22^ within^21^ and between hemispaces.^22^ Risk factors for neglect include right hemisphere injury, middle cerebral artery stroke, temporal injury, parietal injury, increasing age and motoric disability.^23-25^

Neglect is a strong independent predictor of poor prognosis, including poor neurological function and problems with daily tasks.^24,26-29^ Neglect improves in less than half of patients, and recovery is often incomplete.^30^ Almost half of neglect patients show symptoms up to 1-year post-injury,^31^ and 12% of ischaemic stroke survivors show lateralized inattention 7 years post-stroke.^32^

Neglect prevalence estimates vary between 0% and 100% following brain injury, depending on the cohort and assessment.^5,6,33,34^ Recent systematic reviews and meta-analyses of neglect treatments report uncertain evidence of efficacy,^35^ limited-to-no proof of effectiveness,^36^ temporary^37^ or modest long-term benefits,^38^ inadequate methodology^35^ and reporting weaknesses.^39^ Many factors contribute to the lack of reliable treatments and prevalence estimates; one important factor is the limited sensitivity of clinical assessments.^6,33^

### Standard clinical assessments of neglect

Common assessments include pen-and-paper tests and behavioural tests. Pen-and-paper tests include clock drawing, line bisection or cancellation and crossing off search targets among distractors.^40-42^ In clinical practice, performance on these tests is often used to support clinical judgement on the presence of neglect. Behavioural tests assess symptoms within daily activities, such as grooming and locomotion.^43,44^ Behavioural tests are considered more sensitive than pen-and-paper,^6^ which sometimes fail to detect severe neglect.^15^ Nonetheless, pen-and-paper is still widely used in research^12,45-49^ and practice.^50^ Both pen-and-paper and behavioural tests depend on expert opinion and are therefore subjective and susceptible to expectation bias.^12,51^ Neither approach objectively quantifies visuospatial attention in detail across multiple behavioural dimensions (e.g. in orientation of the head, hand and gaze)^17,52-54^ Not all facilities routinely screen for neglect, and only a subset of patients are screened;^50^ thus, broader coverage requires accessible methods.

### Immersive virtual reality assessments of neglect

Computerized assessments can quantify visuospatial attention in multiple dimensions. Among computerized methods, immersive virtual reality (iVR) seems ideal since it provides both gameplay and orientation within the three-dimensional (3D) space, including head, hand and gaze position.^55^ In addition, computerized methods such as iVR allow formalized interpretation of performance which is less reliant on subjective views combined with binary classifiers in pen-and-paper tests.

iVR involves headsets that simulate stereoscopic vision, allowing the player to explore 3D environments.^56^ iVR hand controllers allow interaction with virtual objects. iVR combines experimental control with sensory experiences that simulate and motivate naturalistic behaviour, allowing assessment of multidimensional visuospatial attention. iVR can provide experiences that are safe, engaging, accessible and impossible otherwise.^12,45-49^ Studies have shown that iVR detects and measures neglect in many scenarios, in multiple behavioural dimensions such as gaze and gameplay and with sensitivity comparable with standard assessments.^20,57-70^

### Independent immersive virtual reality neglect classification

Despite progress, standalone iVR neglect classification systems are rare. Previous iVR systems were calibrated assuming the accuracy of standard assessments.^71-75^ Consequently, inaccuracies and biases within standard assessments could also have been reflected in iVR system outputs.^76-78^ To address this, we aimed to develop and validate a standalone iVR neglect classification system.

In the current study, an ‘explainable’^79-81^ mathematical definition of iVR neglect was used. The term ‘explainable’ pertains to the capacity of artificial intelligence systems to offer clear, transparent and interpretable explanations for their decisions. Such clarity is especially crucial in clinical settings, enabling both clinicians and patients to fully grasp and effectively respond to the insights provided by the system. This mathematical definition was based on orientation (to the left or right) and search inefficiency (termed ‘challenge’) to identify patients with neglect, minor atypicality (indicative of inattention not consistent enough to be labelled as neglect) and non-neglect (no atypical behaviour). Neglect was defined as atypical visual search, involving orientation and potential challenge (see Table 1). Atypical visual search, in turn, was defined as gameplay beyond the typical bounds computed via normative modelling^60,82-85^ and provided a means of aggregating multidimensional gameplay.

**Table 1:** iVR classifier

Feature	Category	Explanation	Data source (units)	Feature values
accuracy_subtract	Orientation	Target detection is decreased in one hemispace compared with the other	Accuracy (%)^a^	−1, 0, +1^c^
rt_subtract	Orientation	Target detection speed is decreased in one hemispace compared with the other	RT (s)^a^	−1, 0, +1^c^
headset_mean	Orientation	The headset is oriented towards one hemispace on ≥1 game level	Headset raycast latitude (°)^b^	−1, 0, +1^c^
gaze_mean	Orientation	The eyes are oriented towards one hemispace on ≥1 game level	Gaze raycast latitude (°)^b^	−1, 0, +1^c^
headset_slope	Orientation	The headset orients progressively towards one hemispace as distractors increase	Headset set size slope (°/item)^b^	−1, 0, +1^c^
gaze_slope	Orientation	The eyes orient progressively towards one hemispace as distractors increase	Gaze set size slope (°/item)^b^	−1, 0, +1^c^
accuracy_slope	Challenge	Target detection decreases as the distractor numbers increase; indicative of greater challenge and reduced attentional capacity	Accuracy set size slope (%/item)^b^	0, 1^d^
rt_slope	Challenge	Target detection speed decreases as distractor numbers increase; indicative of greater challenge and reduced attentional capacity	RT set size slope (s/item)^b^	0, 1^d^
Atypicality	Summary	Comprehensiveness of atypical patterns can be labelled as follows:	Features #1 to 8	0 to 8
		Minor atypicality: atypicality ≤ 2		
		Moderate: 2 < atypicality ≤ 4		
		Comprehensive: atypicality > 4		
Orientation	Summary	Comprehensiveness of orientation to one hemispace [<0 left orientation (right neglect), > 0 = right orientation (left neglect)]	Features #1 to 6	−6 to +6
Challenge	Summary	Comprehensiveness of reduced attentional capacity	Features #7 to 8	0, 1, 2
Severity	Summary	A high proportion of target detection errors for the neglected hemispace	Accuracy (%)^a^	0.0 to 1.0
has_neglect	Outcome	Neglect: comprehensive and consistent atypicality including unilateral orientation and potential challenge	Features #9 to 11	Neglect, minor_atypicality, non_neglect
		Minor atypicality: atypicality present but not comprehensive or consistent		
		Non-neglect: no atypicality present		

^a^Pooled across the entire game.

^b^Pooled separately across each set size.

^c^−1, atypical leftward orientation; 0, typical orientation; +1, atypical leftward orientation.

^d^0, typical; 1, atypical.

Hemispace, left or right hemispace; mask, the Boolean (0, 1) indicating whether gameplay was atypical; minor, minor_atypicality.

Selecting the most suitable validation strategy for the iVR system presented a notable challenge. Traditional pen-and-paper methods typically have lower sensitivity than computer-based approaches,^19^ including iVR.^12,45^ Standard validation requires a reference test that can provide definitive ground truth. Therefore, we concentrated on assessing how effectively pen-and-paper methods could detect neglect, using iVR’s neglect identifications as the benchmark for ground truth. We used a multistep validation approach:

1. We investigated the relationship between atypical visual search and risk factors for neglect.
2. We conducted confusion analyses to compare the effectiveness of traditional pen-and-paper methods to identify iVR patient categories (neglect, minor atypicality and non-neglect).
3. We assessed the sensitivity of pen-and-paper techniques in detecting neglect across different iVR neglect categories, defined by the combination of neglected side (left or right), orientation magnitude and challenge magnitude.

**Figure 1:**
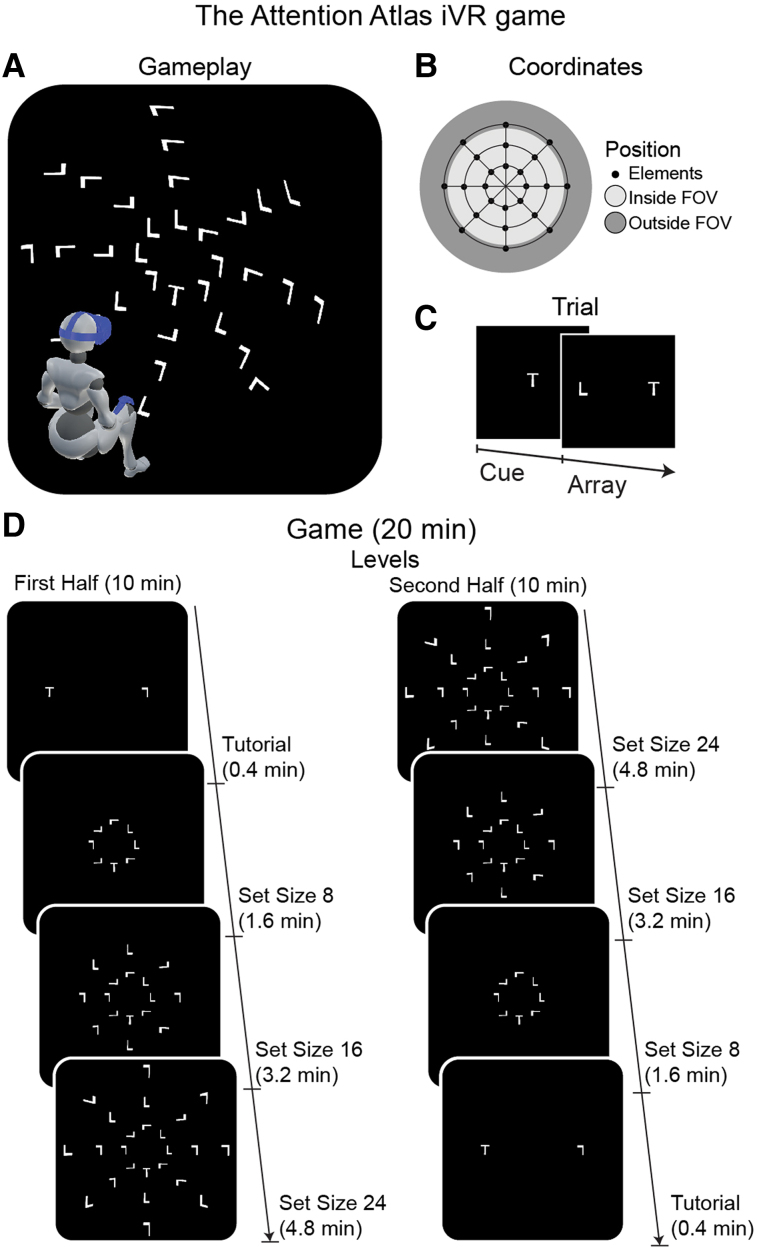
**The Attention Atlas iVR game.** (**A**) Visual search localization gameplay. Array elements were positioned on a spherical surface with an origin at the headset position, calibrated at the start of each level.^55^ This panel depicts the set size of 24 levels. (**B**) Coordinates. Array elements were presented on a spherical grid. Each game level used a subset of possible positions, which could appear within the central field of view (FOV) or towards or beyond its edge, requiring head and eye movements for target localization. For the axes level, the most eccentric horizontal and vertical positions fell outside the central FOV. Note that elements within the FOV depended on the headset position, which changed during the trial as the person moved their head. (**C**) Cue/array trial structure. Cues and arrays were presented until an element was selected. The target was an upright letter ‘T’ among rotated letter ‘L’s’. (**D**) Game. The game comprised 8 levels (2 repetitions of 4 set sizes: 2, 8, 16, and 24 elements). Set sizes in the first and second half were reverse ordered. Each level lasted a fixed minimum duration and terminated upon array element selection. Players were instructed to perform as quickly and accurately as possible and to select a distractor if they could not find the target. Set size of 2 was a tutorial and not included in the analyses. FOV, field of view; iVR, immersive virtual reality; min, minutes.

Controls and brain injury inpatients played The Attention Atlas iVR game.^55,86^ Previously, we showed that this game detects atypical visual search, including neglect.^55^ Participants played a 20 min game where the target letter ‘T’ was among distractors letter ‘L’s’, a stimulus configuration that requires goal-directed visuospatial attention and includes serial spatial reorienting.^87,88^ The number of distractors was varied across levels to calculate set size slope effects as a proxy for attentional capacity^89,90^ (Fig. 1; Video 1). Our primary hypothesis was that, compared with pen-and-paper, iVR would be both more sensitive to and descriptive of neglect.

## Materials and methods

### Ethics approval

The Human Research Ethics Committees of Metro South Health (HREC/2021/QMS/70556), Gold Coast Hospital and Health Service (HREC/2021/QGC/73498) and Griffith University (GU Ref. No: 2021/179) approved the study. Participants provided informed written consent consistent with the Declaration of Helsinki. The experiment was in accordance with hospital and university regulations.

### Participants

We recruited brain injury inpatients from the Neurosciences Rehabilitation Unit at Gold Coast University Hospital (Australia) and the Rehabilitation and Geriatrics Ward at Logan Hospital (Australia). Controls were clinical staff of Gold Coast University Hospital. Recruitment took place between November 2021 and October 2022 and was delayed and hindered by the COVID-19 pandemic.

Patients were eligible if they were clinically stable, had no history of epilepsy, had no reported hemianopia (as discerned by the clinical team member’s examination of the patient’s medical file), had mobility of one or both hands, scored >9 on the University of California Brief Assessment of Capacity to Consent (Gold Coast site only),^91^ were not susceptible to motion sickness [Simulator Sickness Questionnaire (SSQ)^92^ scores < moderate nausea] and had high cognitive functioning of orientation to time and space (Mini-Mental State Examination scores ≥ 6 out of 10).^93^ Controls were required to be unsusceptible to motion sickness (SSQ scores < 3 = moderate nausea).

### Sample size

We aimed for *n* = 50 patients and *n* = 30 controls. Since power analyses were not yet readily available for normative modelling approaches, we based this sample size on frequentist statistics (which were not performed in this study). The desired sample size provided sufficient power (β = 0.92) to detect a large effect (*d* = 0.8) on a two-sided independent-samples *t*-test (α = 0.05) comparing patient and control groups. A large effect size was expected given the sensitivity of computer-based methods demonstrated in previous neglect assessment studies. Finally, we included as many patients as feasibly possible under restraints of hospital access during COVID-19 (*n* = 51) to ensure an adequate number of neglect cases. Fewer controls than patients were deemed sufficient given the expected uniformity of control data.

### Procedure

A clinician team member examined medical files to assess patient eligibility, and patients provided verbal consent for academic team members to undertake recruitment. At Logan Hospital, an experienced neuropsychologist and the academic team (author M.L.) administered three pen-and-paper tests of neglect (see Pen-and-paper assessments of neglect) to patients, and at Gold Coast University Hospital, academic team members administered the pen-and-paper tests. For controls, recruitment was undertaken by an academic team member. Controls were tested on the same day as recruitment. Patients were tested 2 days after recruitment or at the earliest possible time.

For the iVR game, the experimenter first described iVR and the game and informed participants that they were free to discontinue at any time, for any reason. The experimenter fitted the iVR headset. Participants first experienced the aurora night environment of Steam iVR. Eye tracking was calibrated using a five-point scheme. Active gameplay lasted 20 min with breaks between game levels for headset calibration. After the game, participants reported their experiences of iVR on three questionnaires (patients) or four questionnaires (controls) (described in Subjective experiences of iVR).

### Pen-and-paper assessments of neglect

Three tests presented at the patient’s midline. Patients had neglect if indicated on ≥1 test, and the neuropsychologist attributed the results to neglect. CLOX: an executive clock drawing task required patients to draw the numbers and hands on an analogue clock face to read a time of 1:45. Once patients understood and drawing began, we provided no further help.^40^ A preponderance of numbers on either side of the clock indicated neglect. The Single Letter Cancellation Task asked patients to cross out each letter ‘H’ among an array of uppercase letters. Four or more omissions on one half of the page compared with the other indicated neglect. Albert’s Test asked patients to cross out all the lines.^94^ The neuropsychologist illustrated this by crossing out five central lines. If >70% of uncrossed lines were on one half of the page, neglect was indicated.^41^

### Subjective experiences of immersive virtual reality

The SSQ^92^ asked players whether they felt motion sickness during iVR on a seven-point scale (‘no symptoms’–‘vomiting’).

The Game Experience Questionnaire-Revised (GEQ)^95,96^ asked players to indicate gameplay experiences on a 5-point scale (‘not at all’–‘extremely’) on 25 items, loaded onto 4 factors of positive affect, competence, negativity and flow.

The USEQ: A Short Questionnaire for Satisfaction Evaluation of Virtual Rehabilitation Systems^97^ asked patients to rate the iVR’s applicability to their rehabilitation on five items on a five-point scale (‘strongly disagree’–‘strongly agree’).

The System Usability Scale (SUS)^98-100^ asked clinicians to rate iVR hardware usability on 10 five-point (‘strongly disagree’–‘strongly agree’) items. Total scores categorized system usability as acceptable (70–100), marginal (50–70) or unacceptable (0–50).

The Unified Technology Acceptance and Use of Technology Scale (TAS)^101^ asks clinicians to rate iVR’s applicability to their job using 32 items on a 5-point scale (‘strongly disagree’–‘strongly agree’).

### The Attention Atlas

*The Attention Atlas*^55,86^ assessed atypical visual search, which requires search for a single target among distractors (Fig. 1A).^87,88^ Players were seated and used one hand or both hands as preferred. Patients with upper limb paralysis used their more mobile hand. We used spherical coordinates (12.5° radial spacing, 15.0° concentric spacing, 2 m radius) to position elements (Fig. 1B) and an HTC Vive Pro Eye headset (HTC, Taiwan) running on an Alienware Aurora R8 desktop computer (i7 9700, RTX 2070). On each frame, we recorded head, gaze and controller positions for raycast surface intersection and orientation calculation, as previously described.^55,86^ On each trial, we recorded set size, target location, reaction time (RT) and accuracy (correct, target selected; incorrect, distractor selected).

On each trial, a target cue appeared centrally. After the player selected the cue, the search array appeared, and the player located the target (Fig. 1C). The player made selections with the iVR hand controller and virtual laser pointer. We instructed the player to perform as quickly and accurately as possible. If the player could not find the target, we instructed them to select a distractor (recorded as incorrect). The player moved their head, eyes and hand to locate and select the target. Correct target localization was rewarded with confetti at the target’s 3D coordinates. Distractor selection produced no confetti.

The gameplay (20 min) was divided into eight levels and two halves (Fig. 1D). Set size (2, 8, 16 or 24 elements; including 1 target and the remaining distractors) was constant within each level, increased over levels in the first half and decreased in the second half. This provided progression, controlled for time on task and allowed discontinuation at halfway. Levels lasted a fixed minimum duration and ended upon element selection. There was no time-out. The level duration was proportional to set size. The set size of 2 was a tutorial and excluded from analyses. Before each level, we calibrated the headset orientation and position, normalizing element position across players and throughout the game.^55^

### Statistical analysis

#### Subjective experiences of immersive virtual reality

In our analysis of self-reported experiences with iVR, we opted for a descriptive approach rather than inferential statistical analysis. This involved describing median responses on the GEQ and identifying the most common response categories for all other questions. This method provided a straightforward depiction of participants’ experiences with the iVR system.

#### Brain injury patient characteristics

Age and sex were recorded for controls. For patients, we recorded age, sex, days since injury, days in rehab, diagnosis [stroke, traumatic brain injury (TBI), acquired brain injury (ABI)], injured hemisphere (left, right, bilateral), injury region (frontal, temporal, parietal subcortical, white matter, other), stroke type (ischaemic, haemorrhagic, unspecified type), ischaemic stroke artery (middle cerebral artery, posterior cerebral artery, internal carotid artery) and the Functional Independence Measure (FIM).^102^ The FIM assesses independence on 18 activities of daily living on a 7-point scale (‘total assistance’–‘complete independence’) and is composed of two subscales, Motor and Cognition. We expressed FIM on the original 7-point scale by dividing summary scores by the number of items (13 for Motor, 5 for Cognition and 18 for Total).

#### Risk factors for neglect

If the independent iVR classifier is valid, then atypical visual search should correlate with neglect risk factors. We used summary atypicality as the criterion, which was the sum of absolute atypicality across behavioural dimensions (described in Immersive virtual reality neglect classification). We computed univariable Poisson regression models on the patient gameplay with atypical visual search (1–8) as the criterion and patient characteristics as the predictors (pen-and-paper neglect, sex, age, chronicity, independence measures, diagnosis, hemisphere of injury, region of injury, stroke type and ischaemic stroke artery). For each categorical variable, the reference category was chosen as the least frequent category or a combination of the two least frequent categories. Continuous variables were not discretized.

#### Immersive virtual reality neglect classification

##### Overview

The classifier employed normative modelling to detect atypical visual search behaviour, characterized as gameplay that deviates from the norm. We represented the multidimensional aspects of gameplay through eight basic features, categorized as either orientation (directional focus towards the left or right hemispace) or challenge (indicative of search inefficiency, marked by unusually large set size slopes; negative for accuracy and positive for RT). These basic features were then aggregated to form composite orientation and challenge indicators. Based on these indicators, the classifier categorized each participant into one of three groups: neglect (marked by consistent atypicality in both orientation and potentially in challenge), minor atypicality (presence of atypicality but not consistently) and non-neglect (absence of atypical behaviour). Table 1 provides a detailed description of each feature, including the underlying data sources. Through normative modelling, we were able to aggregate multidimensional behaviours onto a common scale, effectively integrating data from various sources.

##### Mathematical neglect definition

Neglect was identified when the orientation value exceeded 1 or when both orientation and challenge exceeded 0. This definition implies that neglect is characterized by either consistent or non-zero orientation, along with supporting evidence of search inefficiency as indicated by the challenge metric. Minor atypicality was recognized in cases where the criteria for neglect were not fully met, yet some level of atypical behaviour was evident. The classification of non-neglect was assigned in the absence of any atypicality.

##### Atypical visual search definition

Atypical visual search was a Boolean variable (0, 1) calculated for each of the ‘orientation’ and ‘challenge’ primitive features. We defined atypical gameplay as:

$$\begin{array}{c} B_{\mathrm{player}}=(\left(M_{\mathrm{player}}<Q1_{\mathrm{controls}}\;\text{–}\;1.5\times \mathrm{IQ}R_{\mathrm{controls}})|(M_{\mathrm{player}}>Q3_{\mathrm{controls}}+1.5\times \mathrm{IQ}R_{\mathrm{controls}}\right))\&\\ (\left(M_{\mathrm{player}}<Q1_{\mathrm{patients}}\;\text{–}\;1.5\times \mathrm{IQ}R_{\mathrm{patients}})|(M_{\mathrm{player}}>Q3_{\mathrm{patients}}+1.5\times \mathrm{IQ}R_{\mathrm{patients}}\right)), \end{array}$$

where *B*_player_ is the Boolean (0, 1) of whether the game was an outlier, *M*_player_ is the game measurement, Q1 is the first quartile, Q3 is the third quartile, and IQR is the interquartile range (IQR) for the specific metric. Atypicality acted to threshold to normalize and combine features and as an overall summary variable.

##### Basic orientation and challenge features

These features were based on four data sources: accuracy (%), RT (s), headset raycast latitude (°) and controller raycast latitude (°). Level means were computed for each set size of distractors and a target (8, 16, 24) to calculate the linear regression slope (i.e. set size effect) for the challenge features and to determine whether any level had an atypically large mean orientation for headset and gaze.

The headset and controller forward vectors cast rays to hit a spherical surface (2 m radius) positioned at the headset origin, calibrated before each game level.^55^ Raycasts were converted from Cartesian (*x*, *y*, z) to spherical coordinates [*r* (fixed 2 m), *θ* (latitude), *ϕ* (longitude)].

Accuracy and RT were pooled across game levels to maximize cell size and to derive subtraction difference scores, contrasting left and right hemispaces and reflecting an orientation to one hemispace or the other. We defined subtraction terms such that positive scores showed a rightward orientation and negative scores showed a leftward orientation. RTs were calculated for correct trials after individual-level outlier removal, performed separately for each level (set size slopes) or trials altogether (subtraction). RTs outliers were defined by:

$$\begin{array}{rl} B_{\mathrm{RT}}\;= & \;(RT_{T}<Q1_{\mathrm{player}}\;\text{–}\;1.5\times \;\mathrm{IQ}R_{\mathrm{player}})|\\ & (RT_{T}>Q3_{\mathrm{player}}+1.5\times \;\mathrm{IQ}R_{\mathrm{player}}), \end{array}$$

where *B*_RT_ is the Boolean of whether the trial RT was an outlier and *T*_RT_ is the trial RT measurement.

##### Summary features

Atypicality was calculated separately for orientation and challenge features and for both combined. Orientation atypicality was summarized by summing basic features, with opposite effects cancelling each other. Additionally, we calculated ‘severity’ as the accuracy in the least accurate hemifield, indicating task difficulty, but it was not used for classification.

##### Classifier heatmaps

We tabulated atypicality in a multidimensional classifier heatmaps [feature × player identifier (ID)]. Player IDs were uniquely assigned and sequentially numbered according to the participation order.

#### Confusion analyses of neglect classification

To explore the relationship between iVR and pen-and-paper neglect classification, we performed standard confusion analyses^103,104^ using iVR as the ground truth reference and pen-and-paper as the predictor.

Confusion matrix analysis is a method used in machine learning to evaluate the performance of classification models. It is presented in the form of a table, which displays the comparison between the actual and predicted values. Key components of a confusion matrix included:

1. True positives (TP): correct positive predictions (both iVR and pen-and-paper tests identify neglect)
2. True negatives (TN): correct negative predictions (both iVR and pen-and-paper tests agree on no neglect)
3. False positives (FP): incorrect positive predictions (iVR does not detect neglect, but pen-and-paper incorrectly does)
4. False negatives (FN): incorrect negative predictions (iVR detects neglect, but pen-and-paper incorrectly does not)

The interplay of TPs, TNs, FPs and FNs reflects the performance of a predictor classifier (pen-and-paper) through metrics such as (i) sensitivity, TP rate; (ii) specificity, TN rate; (iii) balanced accuracy, average of sensitivity and specificity; (iv) positive predictive value, accuracy of positive predictions; (v) negative predictive value, accuracy of negative predictions; (vi) F1 score, harmonic mean of precision and sensitivity; (vii) prevalence, frequency of positive cases in a data set; (viii) detection rate, frequency of correctly identified cases; and (ix) detection prevalence, frequency of positive test results. The formulas for these measures are as follows:

1. Sensitivity = TP/(TP + FN)
2. Specificity = TN/(FP + TN)
3. Balanced accuracy = (sensitivity + specificity)/2
4. Positive predictive value = (sensitivity × prevalence)/((sensitivity × prevalence) + ((1 − specificity) × (1 − prevalence)))
5. Negative predictive value = (specificity × (1 − prevalence))/(((1 − sensitivity) × prevalence) + ((specificity) × (1 − prevalence)))
6. F1 = (1 + β2) × precision × recall/((β2 × precision) + recall), where β = 1 (equal weight given for precision and recall)
7. Prevalence = (TP + FN)/(TP + FP + FN + TN)
8. Detection rate = TP/(TP + FP + FN + TN)
9. Detection prevalence = (TP + FP)/(TP + FP + FN + TN)

We reported standard classification metrics for pen-and-paper, including accuracy and sensitivity for each of the three iVR classes. Since pen-and-paper was binary (neglect, non-neglect), we considered two models: Model 1, a 3 × 2 matrix with all three iVR categories, and Model 2, a 2 × 2 matrix combining minor atypicality and non-neglect categories into a ‘not-neglect’ class. Interrater reliability was calculated using the *κ* statistic, which ranges from +1 to −1 and examines agreement beyond chance, as follows:

$$\kappa \;=\;(P_{o}\;\text{–}\;P_{c})\;/\;(1\;\text{–}\;P_{c}),$$

where *P*_o_ is the probability of agreement and *P*_c_ is probability of chance agreement.

#### Multidimensional neglect categories

We illustrated categories by crossing the three factors of neglected hemispace (right, left), absolute orientation atypicality (high, low) and challenge atypicality (low, high). The high and low values were determined via median split separately for each factor and with both hemispaces included in the median calculation. Thus, this three-factor subtyping scheme results in the logical possibility of eight neglect categories [hemispace (2) × orientation (2) × challenge (2)].

#### Multidimensional neglect atlas

We developed an extensive atlas detailing patterns of unilateral spatial neglect as identified by iVR. This atlas visually synthesizes data from patient interactions and perceptions during visual search tasks, linking attention metrics to spatial neglect’s intensity and presence. It organizes information into rows, each representing a patient category based on neglect severity, from typical to various degrees of impairment. Each row is marked with the neglect type and participant count (*n*). The columns illustrate various gameplay dimensions, showcasing the overall mean (grand M in columns A–E) and mean plus or minus standard error (grand M ± SE in columns F–K) for participants in each category or for individual participants if they are the only member in their category.

The atlas comprises several sections:

- Raycast Attention Maps (columns A–C) display raycast frequency and spatial distribution for a 24-element set. Brighter areas indicate higher attention, with cooler areas denoting lesser attention. Controller raycasts were not used for classification to eliminate incidental orientation from classification. Raycast maps were calculated based on our previous work.^55^
- Gameplay Maps (columns D and E) show accuracy and response time (RT) averages for each target position, using darker shades to highlight inattention.
- Orientation Features (columns F–I) include mean accuracy and RT differences for right versus left targets (columns F and G), alongside raycast averages across various set sizes (columns H and I).
- Challenge Features (columns J and K) present accuracy and RT variations by set size.

An asterisk in columns F–K denotes atypical visual search patterns in one or more patients per category.

#### Statistical software

Statistical analyses were performed in Python 3.10.6 and R 4.1.1.

## Results

### Participant characteristics

Fifty-one brain injury patients (mean age = 65.5 years, SD = 17.3 years; *n* = 37 males) and 30 controls (mean age = 40.5 years, SD = 12.5; *n* = 11 males) (for the cohort definition flowchart, see Supplementary Fig. 1) experienced the VR.

### Subjective experiences of immersive virtual reality

Of the patients, 38 completed the Game Experience survey, 40 completed the Satisfaction Evaluation of Virtual Rehabilitation Systems, and 48 completed the motion sickness survey. Among controls, only one did not complete the SUS, and one other did not complete the Technology Acceptance scale.

Both patient and control groups reported favourable experiences with iVR, as detailed in Supplementary Figs. S2 and S3:

1. Motion Sickness (SSQ): minimal issues were reported, with most participants experiencing no symptoms or only rare stomach awareness (Supplementary Figs. 2A and 3A).
2. Game Experience (GEQ): the overall response was positive. Median scores for flow, competence and positive affect were moderate to fair, while negative affect scores were minimal, indicating low levels of negativity (Supplementary Figs. 2B and 3B).
3. System Relevance and Comfort (USEQ): patients generally agreed or strongly agreed that the iVR system was relevant for their rehabilitation and disagreed with experiencing discomfort. Conversely, control participants rated the system’s usability as acceptable or marginal (SUS) (Supplementary Fig. 2C), with neutral to positive responses to items such as ‘I like working with the system’ and ‘working with the system is fun’ (TAS) (Supplementary Fig. 2D).

These results reinforce previous research, highlighting the potential of iVR in neurorehabilitation contexts.^20,57-70^

### Brain injury patient characteristics

Table 2 presents brain injury patient characteristics. Mean summary atypical visual search—iVR gameplay beyond the typical bounds—was 1.3 ± 2.0 SD, indicating substantial atypicality overall. *N* = 9 (17.7%) patients had pen-and-paper neglect. Patients were recruited on average 43.9 days post-injury, having spent 18.9 days at inpatient neurorehabilitation. On average, patients scored 3.4 (moderate assistance) for motoric tasks and 5.1 (supervision) for cognitive tasks, indicating considerable disability. Stroke was the most common diagnosis (74.5%), followed by other ABI (15.7%) and TBI (9.8%). Left hemisphere injuries were most common (40.0%), followed by right hemisphere (38.0%) and bilateral injuries (22.0%). Injured regions were broadly categorized non-exclusively as cortical (frontal, temporal, parietal), subcortical and white matter. Most patients (84.3%) presented with injuries that encompassed multiple regions. Cortical injuries were most common (82.4%), followed by subcortical (51.0%) and white matter injuries (33.3%). A total of 15.7% of patients had injuries to ‘other’ brain regions. Stroke injuries most commonly were ischaemic and involved the middle cerebral artery.

**Table 2:** Risk factors for visuospatial atypicality

Patient characteristic	Total, *N* = 51 (100.0%)	IRR (95% CI)	P^a^
VR atypicality	1.3 ± 2.0		
Pen-and-paper neglect presence			
0	42 (82.4)	Ref.	
1	9 (17.6)	7.18 (4.41–11.90)	<0.001
Sex			
Male	36 (70.6)	Ref.	
Female	15 (29.4)	1.04 (0.60–1.74)	0.87
Age in years	65.4 ± 17.4	1.03 (1.01–1.04)	0.002
Chronicity			
Days since injury	43.9 ± 50.0		
Days in rehab	18.9 ± 34.2	0.97 (0.94–0.99)	0.02
Functional Independence Measure			
FIM Motor	3.4 ± 1.6	0.63 (0.51–0.77)	<0.001
FIM Cognition	5.1 ± 1.3	0.87 (0.71–1.08)	0.21
FIM Total	3.9 ± 1.1		
Diagnosis			
Stroke	38 (74.5)	3.42 (1.61–8.86)	0.004
Other ABI	8 (15.7)	Ref.	
TBI	5 (9.8)	Ref.	
Injury hemisphere			
Left	20 (40.0)	1.60 (0.66–4.46)	0.33
Right	19 (38.0)	4.63 (2.13–12.12)	<0.001
Bilateral	11 (22.0)	Ref.	
Injury region			
Frontal	16 (31.4)	0.12 (0.05–0.28)	<0.001
Temporal	14 (27.5)	2.37 (1.25–4.39)	0.007
Parietal	12 (23.5)	3.74 (1.80–7.47)	<0.001
Subcortical	26 (51.0)	0.46 (0.26–0.78)	0.005
White matter	17 (33.3)	0.79 (0.41–1.46)	0.46
Other region	8 (15.7)		
Stroke type			
Ischaemic	31 (60.8)	0.27 (0.14–0.54)	<0.001
Haemorrhagic	6 (11.8)	0.80 (0.38–1.73)	0.56
Unspecified type	2 (3.9)	Ref.	
Ischaemic stroke artery			
MCA	18 (35.3)	5.27 (2.00–18.23)	0.002
PCA	4 (7.8)	1.25 (0.29–3.64)	0.72
ICA	1 (2.0)	Ref.	
Other artery	13 (25.5)	Ref.	

*Note.* Categorical values are given in *n* (%), and continuous values are given in M ± SD.

IRR, incidence rate ratio; MCA, middle cerebral artery; PCA, posterior cerebral artery; ICA, internal carotid artery.

^a^*P* value of a Poisson regression with atypicality (0–8) as the predictor.

### Risk factors for neglect

Outlier cut-offs are provided in Supplementary Table 1. Table 2 presents the univariable Poisson regression model results with patient characteristics as predictors and atypical visual search—as the criterion. Grand mean atypical visual search was 1.3 ± 2.0 SD, indicating substantial atypicality within the sample. The strongest risk factor for iVR defined atypical visual search was pen-and-paper neglect, which was significantly associated with increased log odds of atypicality with an incidence rate ratio (IRR) of 7.18 (95% CI, 4.41–11.90); thus, the point estimate predicted a 7.2-fold or 620% increase in atypicality compared with the absence of pen-and-paper neglect.

Consistent with the pen-and-paper association, risk factors of neglect^23-25^ were significantly associated with increased atypicality. From highest to lowest, the risk factors were MCA stroke (IRR, 5.27, 95% CI 2.00–18.23), right hemisphere injury (IRR, 4.63, 95% CI 2.13–12.12), parietal injury (IRR, 3.74, 95% CI 1.80–7.47), stroke (IRR, 3.42, 95% CI 1.61–8.86) and temporal injury (IRR, 2.37, 95% CI 1.25–4.39). From highest to lowest, the protective factors were frontal injury, ischaemic stroke, subcortical injury and motoric independence on activities of daily living (IRR, 0.63, 95% CI 0.51–0.77).

### Immersive virtual reality neglect classification

**Figure 2:**
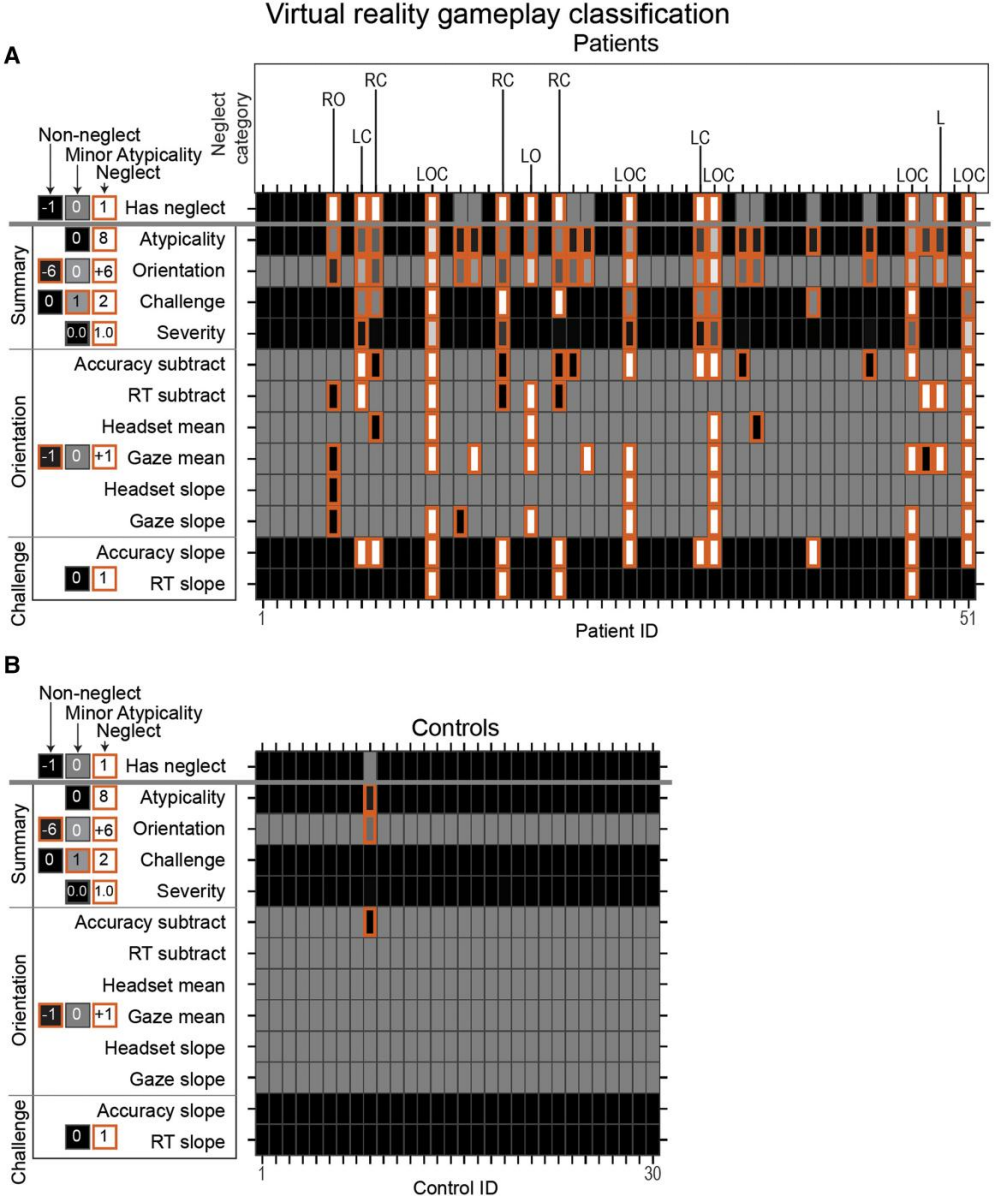
**Heatmaps displaying multidimensional classifier outcomes.** This figure contrasts atypical visual search patterns in (**A**) patients and (**B**) controls, using orange or thick-bordered squares to highlight such patterns across summary, orientation and challenge features. The top row specifically denotes iVR-identified neglect cases with orange or thick borders, minor atypicalities with grey fill and typical patterns with black fill. Feature intensities are normalized: black signifies low, white high and grey intermediate levels. The legend to the left details the non-normalized value ranges, with orientation values interpreted as negative for leftward and positive for rightward tendencies. Challenge metrics are represented by positive integers. Atypical visual search, determined through normative modelling, identifies gameplay deviations beyond the 1.5 × IQR cut-offs for both patients and controls, detailed in Supplementary Table 1. Orientation and challenge metrics are quantified into directional and positive integers, respectively. In panel **A**, iVR neglect cases are indicated by their neglect category. L, left; LC, left challenge; LO, left orientation; LOC, left orientation challenge; RC, right challenge; RO, right orientation; RT, reaction time.

Figure 2 presents the classifier heatmaps for patients (Fig. 2A) and controls (Fig. 2B). These depict individuals as rows and features as columns, with orange-highlighted cells indicating atypical visual search. As expected, atypicality was more prevalent for patients than controls (cf. Figure 2A and B).

Of the patients, the classifier labelled *n =* 13 (25.5%) with neglect (summary atypicality of M = 4.3 ± 1.7 SD), which is indicated by ‘1’ in the first column of Fig. 2A. The classifier labelled *n* = 9 patients (17.6%) with minor atypicality (atypicality M = 1.1, ± 0.3 SD) and the remaining 29 patients (56.9%) as non-neglect.

Of the neglect-labelled patients, *n =* 9 (17.7%) had left neglect (orientation M = 3.6, ± 1.7 SD), eight of whom had right hemisphere injuries and one of whom had bilateral injuries; *n =* 4 (7.8%) had right neglect (orientation M = −2.5 ± 1.0 SD), three of whom had left hemisphere injuries and one of whom had a right hemisphere injury; *n =* 10 (76.9%) were labelled with challenge (i.e. reduced attentional capacity indexed by set size; M = 1.4, ± 0.5 SD); and *n =* 8 (15.7%) showed significant severity, a high proportion of target detection errors for the neglected hemispace (severity M = 0.4, ± 0.3 SD).

### Confusion analyses of neglect classification

Table 3 presents the confusion matrix and classification statistics with iVR as the ground truth and pen-and-paper as the predictor. The ‘All’ marginal column and row indicate iVR and pen-and-paper classes, and the cells indicate their cross-tabulation.

**Table 3:** Confusion analyses of neglect classification

	iVR classes, *n* (%)							
Pen-and-paper, *n* (%)	Neglect	Non-neglect	Minor atypicality	All	Accuracy (%) (95% CI)^a^	Accuracy null (%)^b^	*P* null	*κ*
Neglect	7 (13.7)	1 (2.0)	1 (2.0)	9 (17.6)				
Non-neglect	6 (11.8)	28 (54.9)	8 (15.7)	42 (82.4)				
Minor atypicality	0 (0.0)	0 (0.0)	0 (0.0)	0 (0.0)				
All	13 (25.5)	29 (56.9)	9 (17.6)	51 (100.0)				
Model 1^c^					68.6 (54.1–80.9)	56.9	0.06	0.4
Model 2^d^					84.3 (71.4–93.0)	74.5	0.07	0.5
Sensitivity^e^	53.8	96.6	0.0					
Specificity^f^	94.7	36.4	100.0					
Balanced accuracy^g^	74.3	66.5	50.0					
Positive predictive value^h^	77.8	66.7						
Negative predictive value^i^	85.7	88.9	82.4					
F1^j^	63.6	78.9						
Prevalence^k^	25.5	56.9	17.6					
Detection rate^l^	13.7	54.9	0.0					
Detection prevalence^m^	17.6	82.4	0.0					

TP, true positives; TN, true negatives; FN, false negatives; FP, false positives.

^a^Accuracy = (TP + FN)/(TP + FP + FN + TN).

^b^Expected accuracy of always guessing the most prevalent class (not-neglect, full model; non-neglect, reduced model).

^c^Uses all three AI classes (neglect, non-neglect, minor atypicality).

^d^Pools AI non-neglect and minor atypicality classes into a single non-neglect class. Note that AI neglect class statistics are equivalent for the reduced and full model.

^e^Sensitivity = TP/(TP + FN).

^f^Specificity = TN/(FP + TN).

^g^Balanced accuracy = (sensitivity + specificity)/2.

^h^Positive predictive value = (sensitivity × prevalence)/((sensitivity × prevalence) + ((1 − specificity) × (1 − prevalence))).

^i^Negative predictive value = (specificity × (1 − prevalence))/(((1 − sensitivity) × prevalence) + ((specificity) × (1 − prevalence))).

^j^F1 = (1 + β^2^) × precision × recall/((β^2^ × precision) + recall); β = 1 (equal weight given for precision and recall).

^k^Prevalence = (TP + FN)/(TP + FP + FN + TN).

^l^Detection rate = TP/(TP + FP + FN + TN).

^m^Detection prevalence = (TP + FP)/(TP + FP + FN + TN).

For Model 1, which included all 3 iVR classes, pen-and-paper had 7 TPs and 6 FNs for neglect, 28 TPs and 1 FN for non-neglect and 0 predictions for minor atypicality. Six FNs were for patients who showed substantial atypicality overall (Patients 10, 12, 13, 51, 61, and 78; M = 3.0 ± 0.9 SD; Fig. 2). The overall accuracy of pen-and-paper was 68.6% (95% CI = 54.1–80.9), which was not significantly different from null/chance accuracy of 56.9% (*P* = 0.06)—always guessing the most prevalent class (non-neglect). The interrater reliability was ‘slight’ (*κ* = 0.4).^105^ This finding suggests poor agreement, with discrepancies in neglect-positive classification, which is expected given the poor sensitivity of pen-and-paper and the hypothesis that iVR is more sensitive to identifying cases of neglect. As we will continue to describe and discuss, this slight interrater reliability is best explained by iVR detecting neglect with greater sensitivity and in greater detail than is possible with pen-and-paper.

For Model 2, which combined iVR non-neglect and minor atypicality classes into a single ‘not-neglect’ class, pen-and-paper had six TPs and two FPs, which pen-and-paper labelled as right neglect. One FP was iVR non-neglect (Patient 57; atypicality = 0), and the other was iVR minor atypicality (Patient 77; atypicality = 2). This latter patient was right-oriented on RT but left-oriented on gaze mean and thus did not show consistent iVR orientation (sum = 0) or any challenge (Fig. 2). Relative to Model 1, the accuracy (84.3%, 95% CI = 71.4–93.0) and interreliability (‘moderate’, *κ* = 0.5) was improved; however, pen-and-paper’s accuracy still did not differ significantly from null accuracy (*P* = 0.07).

For iVR neglect, pen-and-paper had poor sensitivity, correctly labelling only 53.8% of neglect cases, but high specificity, correctly labelling 94.7% of ‘not-neglect’ cases as non-neglect (non-neglect and minor atypicality combined). Hence, the balanced (i.e. arithmetic) mean accuracy was 74.3%. The positive predictive value indicated that 77.8% of pen-and-paper neglect were iVR neglect, and the negative predictive value indicated that 85.7% of pen-and-paper non-neglect cases were iVR ‘not-neglect’. The F1 score (i.e. harmonic mean accuracy) was relatively low (63.6%), reflecting the penalty imposed for pen-and-paper’s imbalance between low sensitivity and high specificity. The overall prevalence of iVR neglect was 25.5%, but the pen-and-paper neglect detection rate was only 13.7%. The detection prevalence rate was marginally higher at 17.6% due to two FP pen-and-paper predictions.

For iVR non-neglect (computed under Model 1), pen-and-paper had high sensitivity, correctly labelling 96.6% of iVR non-neglect cases as non-neglect, but poor specificity, incorrectly labelling 36.4% of iVR neglect and minor atypicality cases as non-neglect. The overall prevalence of iVR non-neglect was 54.9%, yet pen-and-paper assigned non-neglect in 82.4% of cases. For the iVR minor atypicality class, which had a 17.6% prevalence, pen-and-paper had 0.0% sensitivity.

In summary, these results suggested that pen-and-paper has poor accuracy, with low sensitivity but high specificity for iVR neglect, high sensitivity but low specificity for non-neglect and insensitivity to iVR minor atypicality.

### Multidimensional neglect categories

Table 4 presents neglect categories based on median splits and crossing of neglected hemispace, orientation and challenge factors. We adopted the following three-character codes (e.g. ‘LOC’): the first character indicated the neglected hemispace (‘L’ = left, ‘R’ = right), the second character (‘O’) indicated above-median orientation, and the third character indicated above-median challenge (‘C’). The last two characters were omitted for below median scores.

**Table 4:** Multidimensional neglect categories

Subtype^a^	*N* = 13 (100.0%)	Detected on pen-and-paper (%)	Atypicality (0 to 8)	Orientation (−6 to +6)	Challenge (0 to 2)	Severity (0.00 to 1.00)
Neglect LOC	5 (38.5)	100.0	6.0 ± 1.0	4.6 ± 1.1	1.4 ± 0.5	0.49 ± 0.31
Neglect LO	1 (7.7)	100.0	4.0	4.0	0.0	0.00
Neglect LC	2 (15.4)	0.0	2.0 ± 0.7	1.5 ± 0.7	1.0 ± 0.0	0.09 ± 0.00
Neglect L	1 (7.7)	0.0	2.0	2.0	0.0	0.00
Neglect RO	1 (7.7)	0.0	4.0	−4.0	0.0	0.02
Neglect RC	3 (23.1)	33.0	3.7 ± 0.6	−2.0 ± 0.0	1.7 ± 0.6	0.10 ± 0.10
Non-neglect	29		0.0 ± 0.0	0.0 ± 0.0	0.0 ± 0.0	0.00 ± 0.01
Minor atypicality	9		1.1 ± 0.3	−0.3 ± 0.9	0.1 ± 0.3	0.02 ± 0.02

Median cut-offs: ‘O’, |orientation| ≥ 3.0; ‘C’, challenge ≥ 1.0. Values given in M ± SD.

^a^‘L’, left hemispace neglect; ‘R’, right hemispace neglect; ‘O’, above-median orientation; ‘C’, above-median challenge. Omission of ‘O’ and/or ‘C’, below median orientation and/or challenge.

Of eight possible categories, we identified six within this sample: all four left neglect categories: neglect LOC (*n* = 5), neglect LO (*n* = 1), neglect LC (*n* = 2), and neglect L (*n* = 1), and two of four right neglect categories, neglect RO (*n* = 1) and neglect RC (*n* = 3). The most prevalent category was also the most visuospatially atypical: neglect LOC, with high atypicality (M = 6.0 ± 1.0 SD), high rightward orientation (M = 4.6 ± 1.1 SD), high challenge (M = 1.4 ± 0.5 SD) and high severity (M *=* 0.49 ± 0.31 SD). The next most prevalent category was neglect RC with moderate atypicality (M = 3.7 ± 0.6), minor leftward orientation (M = −2.0 ± 0.0 SD), consistent challenge (M = 1.7 ± 0.6 SD) and low severity (M = 0.10 ± 0.10 SD). Pen-and-paper detected five of five LOC, 1 of 1 LO and 1 of 3 RC. Pen-and-paper failed to detect the other categories (LC, L and RO). By iVR neglected hemispace, pen-and-paper detected six of nine (66.6%) left neglect cases and one of four right neglect cases (25%).

### Multidimensional neglect atlas

**Figure 3:**
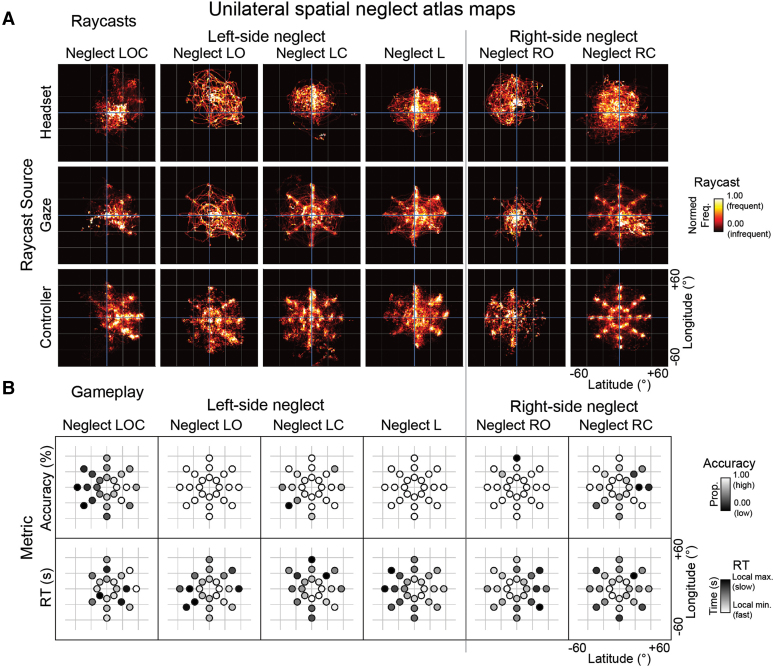
**Multidimensional neglect atlas maps.** This figure visualizes attentional patterns associated with different neglect categories, all from a first-person viewpoint. (**A**) Raycast visualization: These maps synthesize headset, controller and gaze data into raycast diagrams, which function as two-dimensional histograms with a 1° bin width, tailored for set size = 24 level trials. They colour-code attention frequency: high-frequency areas in whites and yellows signify areas most attended to, areas in reds indicate lesser attention, and black areas were not attended. (**B**) Gameplay performance maps. Here, accuracy and RT metrics from gameplay are mapped by target location, each metric with its own shading scale. Lighter shades represent higher accuracy or quicker RTs (indicating better performance), while darker shades denote lower accuracy or slower RTs (poorer performance). RT data are included only for correct responses, after removing outliers. Consequently, some data points are absent for neglect groups with left challenge (LC), left orientation (LO) and left orientation challenge (LOC). Freq., frequency; L, left; LC, left challenge; LO, left orientation; LOC, left orientation challenge; max., maxima; min., minima; Prop., proportion; RC, right challenge; RO, right orientation; RT, reaction time.

**Figure 4:**
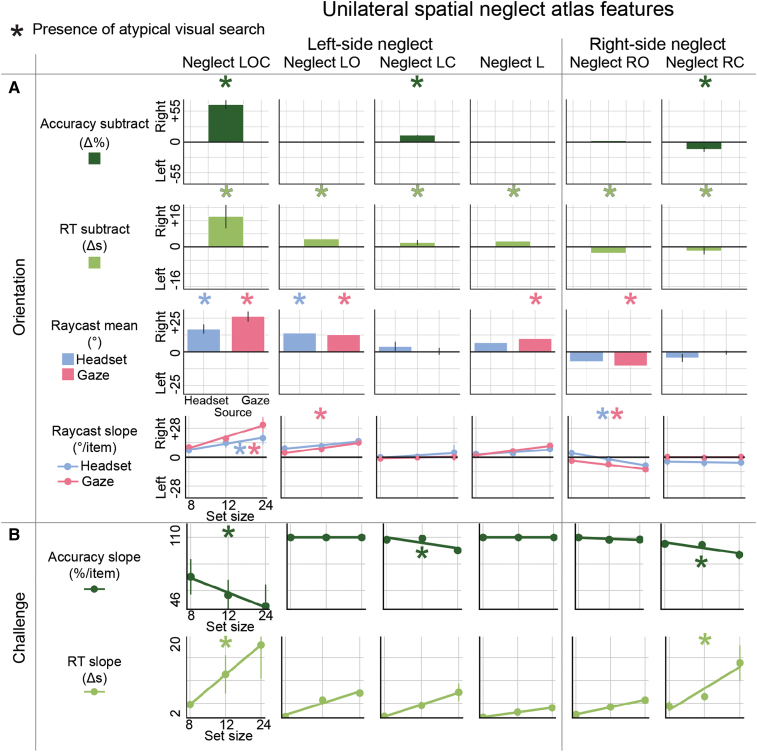
**Multidimensional neglect atlas features.** This figure synthesizes attentional patterns into aggregate summary statistics, illustrating the basic elements of orientation and challenge responses across neglect categories. (**A**) Orientation. Demonstrated here are the orientation features, where positive values signify a rightward orientation and negative values indicate a leftward orientation. (**B**) Challenge. Results are plotted from the grand minima to grand maxima, separately for RT and accuracy. Asterisks indicate the presence of atypical features among most members of each category with multiple individuals. For categories with only one member, asterisks highlight any deviation from typical patterns for that individual. Atypical visual search, as identified through normative modelling, refers to gameplay that deviates from established norms for both patients and controls, with these deviations quantified as exceeding 1.5 times the IQR. Such cut-offs, detailed in Supplementary Table 1, underpin the transformation of raw scores into directional integers for orientation analyses and positive integers for evaluating challenge responses. L, left; LC, left challenge; LO, left orientation; LOC, left orientation challenge; RC, right challenge; RO, right orientation; RT, reaction time; s, seconds.

The study introduces a multidimensional neglect atlas, delineated in Figs 3 and 4, which document various categories of neglect, each in a distinct column. Figure 3 presents visualizations of attention maps, employing raycast data from multiple sources—headset, gaze and controller—and gameplay performance maps of accuracy and RT, for searches of set size 24. Figure 4 explores the directional bias away from the neglected space and examines how distinct neglect categories respond to escalating task demands, facilitating a comparative analysis of neglect manifestations within different facets of iVR gameplay.

In Fig. 3A, the distinct orientations and intensities of neglect across categories are depicted through raycast maps. These maps, characterized by varying attentional focus, illustrate the distribution of attention across space. Notably, left-side neglect categories (LOC, LO, LC and L) predominantly attend to the right side, showing an inattention to the left, while right-side categories (RO and RC) exhibit the reverse pattern. This attentional preference is also reflected in the gameplay (Fig. 3B), where neglect influences both accuracy and RTs, with varying patterns across categories.

Delving into specifics, neglect LOC stands out with a stark rightward attentional bias, mirrored in gameplay data by an accuracy gradient favouring right-side targets. Conversely, neglect LO and L, while less pronounced overall, still show a rightward focus in raycast maps and faster RTs for right-side stimuli. Neglect LC’s unique pattern is less overt in raycast maps but instead is evident in gameplay, with lower accuracy and slower RTs for left-side targets. The right-side neglect categories exhibit their own distinctive patterns, with neglect RO and RC differing in their impact on RTs and accuracy.

Figure 4’s analysis further illustrates the variability in how these neglect categories manifest in orientation (Fig. 4A) and challenge features (Fig. 4B), with a notable distinction between these two facets. Figure 4A suggests that RT disparities between left and right space are a consistent marker of neglect, while accuracy disparities are less predictable but consistently signal the co-occurrence of accuracy challenge effects. Raycast analyses indicate that neglect tended to manifest more commonly in gaze than headset orientation effects. The magnitude of orientation effects did not always correspond to the presence of challenge effects. For instance, both neglect LC and RC showed no group majority presence of raycast effects and yet showed reduced accuracy for higher set sizes.

These results provide a comprehensive atlas of neglect-related attentional categories. Analogous results for non-neglect patients, which show more typical visual search overall, are depicted in Supplementary Figs. 4 and 5. These results illustrate the varied impacts of neglect on orientation and challenge, providing a nuanced view of how different neglect types can manifest within iVR gameplay.

## Discussion

We report an explainable iVR neglect classifier that was independent of standard assessments and clinical data. The classifier labelled 13 patients (25.5%) with neglect, including 9 patients (17.7%) with left neglect and 4 patients (7.9%) with right neglect. The iVR neglected hemispace was contralateral or bilateral to the injury in 92.3% of cases, consistent with contralesional definitions of neglect.^1-4^ The classifier determined that 9 patients (17.6%) had minor atypicality, and the remaining 29 patients (56.9%) had non-neglect. The classifier used features based on atypical visual search, defined as normatively modelled atypical gameplay, and atypical visual search was positively associated with neglect risk factors including pen-and-paper neglect, right hemisphere injury, stroke, middle cerebral artery stroke, temporal injury, parietal injury and motoric disability.^23-25^ Consistent with our hypothesis that iVR would be more sensitive to and descriptive of neglect than pen-and-paper, confusion analyses showed that pen-and-paper’s accuracy, sensitivity and specificity were insufficient for iVR neglect classification. iVR independently categorized neglect and described its multidimensionality.

A barrier for neglect classifiers has been the inability to quantify, threshold and categorize multidimensional gameplay. Normative modelling achieved this by thresholding features on to the common scale of atypical visual search, independent of the behavioural dimension, providing an explainable mathematical definition of neglect. Although normative modelling is common in fields such as psychiatry and psychology,^82-85^ it is rare in iVR neglect assessment^60^ and has not informed neglect classification.

An explainable mathematical definition of neglect underpinned classification. Neglect was defined as comprehensive atypical visual search, involving orientation and potential challenge (i.e. attentional capacity limits; Table 1). Neglect-positive classification required that the overall atypicality score was ≥2, therefore providing converging evidence across features. This threshold for neglect-positive classification was met via one of two means: by orientation alone or by both orientation and challenge. Only Patient 61 was labelled neglect using both orientation and challenge; the remaining 12 iVR neglect cases were labelled by orientation alone. Of these patients, eight showed challenge ≥ 1, indicating occurrence of orientation and challenge. This result is consistent with the idea that attention capacity limits can be characteristic of neglect.^15,18-25,106,107^

We validated iVR neglect classification with confusion analyses. We used iVR as the ground truth and pen-and-paper as the predictor, an approach justifiable on the grounds that pen-and-paper is insensitive and therefore does not provide a reliable reference compared with computerized methods^19^ including iVR.^12,45^ Overall, pen-and-paper performed poorly in classifying iVR neglect. For both Model 1 (including all iVR classes) and Model 2 (with a single ‘not-neglect’ class), pen-and-paper produced accuracy not significantly different to always guessing ‘non-neglect’. Pen-and-paper had relatively poor sensitivity for iVR neglect, correctly labelling only 53.8% of neglect cases, but good specificity, correctly labelling 94.7% of ‘not-neglect’ cases as non-neglect. Conversely, Model 1 showed that pen-and-paper had high sensitivity for iVR non-neglect, correctly labelling 96.6% of non-neglect cases as non-neglect, but poor specificity, correctly labelling 36.4% of iVR neglect and minor atypicality as ‘not’ non-neglect. This latter result reflected a combination of both poor sensitivity for neglect and 0% sensitivity for minor atypicality. Usually, pen-and-paper provides two rather than three classes; all neglect cases are equivalent, and there is no state between neglect and non-neglect. As the iVR results suggest, there are subthreshold neglect-consistent atypicality patterns, here termed ‘minor atypicality’, which can be distinguished from typical attention.

iVR neglect classification illustrated the multidimensionality of neglect both between and within patients. Categorization revealed that the most prevalent classes were neglect LOC and neglect RC. Neglect LOC was more common and had greater orientation atypicality than neglect RC and greater challenge and severity. Overall, left neglect (69.2% of cases) was more common than right neglect (30.8% of cases), consistent with previous research.^5,6,33,34^ However, right neglect was common and associated with challenge, illustrating the need for objective neglect assessments that encompass both hemispaces. Pen-and-paper detected all neglect LOC cases, but only 66.6% of all iVR detected left neglect cases, and 25% of all iVR detected right neglect cases. Thus, pen-and-paper was insensitive to more mild neglect and right neglect.

Notably, one patient demonstrated right-side neglect following a right hemisphere injury, a finding at odds with typical spatial neglect patterns. In rare and complex cases, such ipsilesional neglect can occur due to factors like specific brain region involvement, additional neurological conditions and compensatory mechanisms.^108,109^ Notably, a study reported ipsilesional spatial bias after a focal cerebral infarction in the medial agranular cortex,^110^ which includes the frontal eye fields,^111^ a key area for attention control. This aligns with our patient’s condition, who had a tumour resected from the frontotemporal region encompassing the agranular cortex. These findings underscore the need for further research to unravel the intricate relationships between injury specifics, aetiology, recovery processes and the manifestation of neglect symptoms, including their laterality.

### Limitations

Prevalence estimates of neglect vary widely, between 0% and 100%.^5,6,33,34^ Our sample differs from previous iVR studies, which tended overall to focus on chronic right hemisphere injuries.^20,57-70^ Instead, we investigated non-acute patients, including non-stroke injuries and left hemisphere and bilateral injuries. Regardless, we tested only a subset of inpatients: those with the capacity to consent, with high cognitive function, without motion sickness susceptibility, without hemianopia and with intact mobility of one or both hands. Thus, the 25.5% found to have neglect in this study may represent selection bias and a conservative estimate of iVR neglect prevalence and severity. To accurately estimate prevalence, it will be necessary to map the time course^31^ from acute injury to discharge and beyond using sensitive and objective methods.

Control and patients were not matched on age and sex. This may have resulted in exaggerated differences between controls and patients. An older control group would likely have displayed more diversity and atypicality. However, these factors were unlikely to have significantly affected neglect classification and the observation of increased atypicality among patients compared with controls would have remained. First, outlier cut-offs were determined using both patients and control data. Second, individual-level performance was based on difference scores and set size effects rather than absolute measurements. Nonetheless, to better derive healthy population norms, we recommend that future studies more closely match controls and patients on person-level characteristics.

Finally, an increase in sample size would allow more precise knowledge of the bounds of the classifications and cut-offs. The accessibility of immersive VR technology makes gaining a larger sample through large multi-site studies far more feasible than in the past. In addition, a larger sample might allow for greater confidence in the generalizability of the results; however, we believe that the participants are representative of the general population of brain injury inpatients. However, we do recommend replication of our results at different test sites with larger samples.

## Conclusion

We have designed an iVR neglect classifier that effectively detects pervasive visuospatial anomalies. It elucidates clear and understandable neglect categories, many of which are often overlooked or inadequately described by traditional pen-and-paper methods. Consequently, the iVR classification not only surpasses the sensitivity and descriptiveness of pen-and-paper approaches but also operates independently from existing tests. However, for iVR and other methods to be effective in practice, both usability testing and implementation science, a discipline concerned with applying research in practice, are needed. iVR can be fun, engaging, low-risk and more sensitive than pen-and-paper. Further progress requires consultation with clinicians, patients and other stakeholders to translate technological solutions into effective real-world applications that advance understanding and improve rehabilitation outcomes.

## Supplementary Material

fcae145_Supplementary_Data

## Data Availability

Access to the data and source code is available upon request and following discussion with Griffith University. The data are not publicly available due to ethical restrictions (to ensure the anonymity of participants) and due to ongoing commercialization discussions (see ‘Competing interests’).
