# Supplementary material for: Virtual reality gameplay classification illustrates the multidimensionality of visuospatial neglect
Source: Brain Commun. 2024 May 3;6(4):fcae145. doi: 10.1093/braincomms/fcae145 (PMC11333965; doi:10.1093/braincomms/fcae145)
Supplement: fcae145_Supplementary_Data [file fcae145_Supplementary_Data.docx]

**Supplementary Figure 1 Cohort definition flowchart**

**
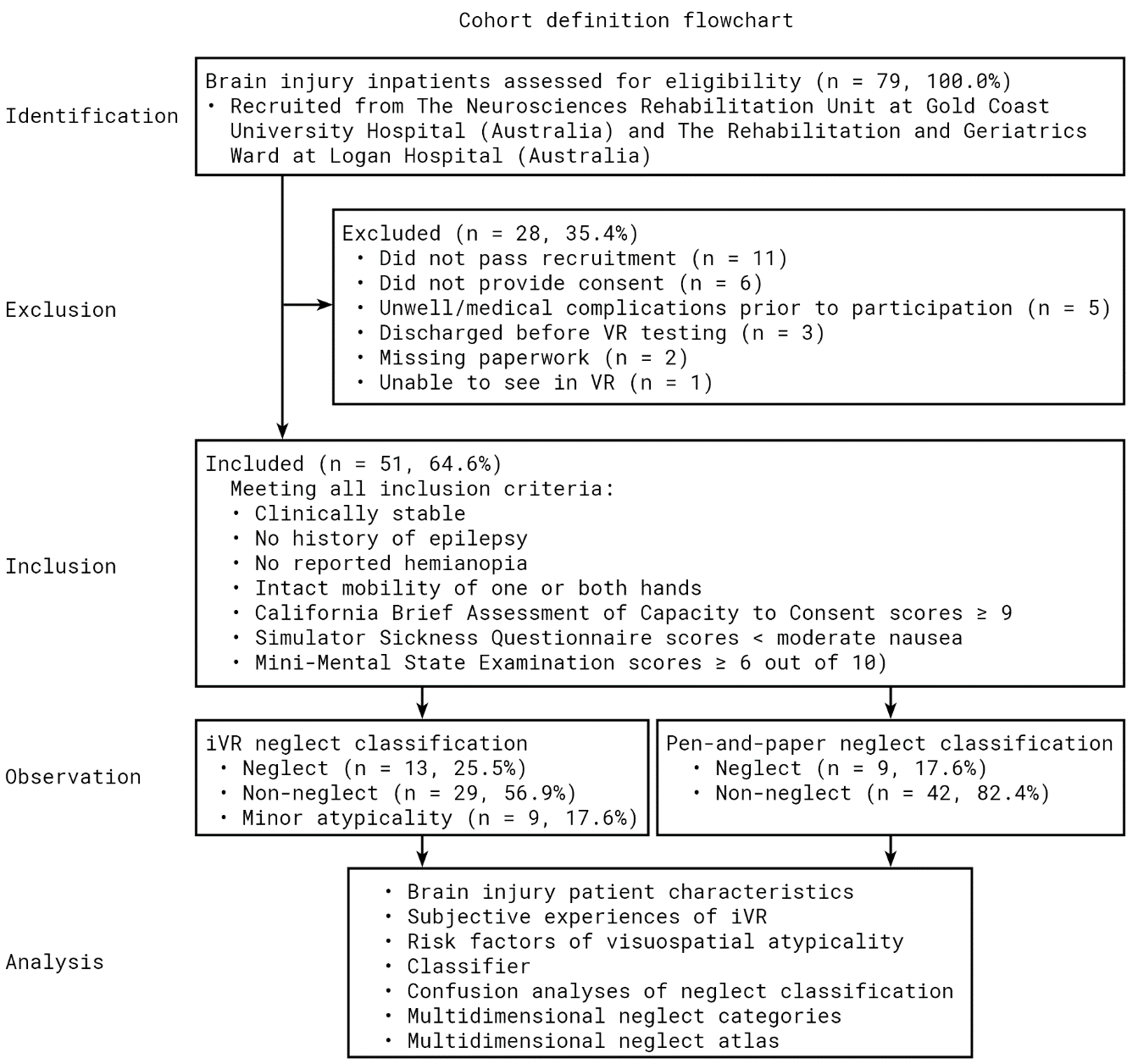
**

**Abbreviations: iVR, immersive virtual reality.**

**Supplementary Table 1 Outlier cutoffs**

| Feature | Lower cutoff | Upper cutoff |
| --- | --- | --- |
| accuracy_subtract | -2.37 | 2.73 |
| rt_subtract | -1.62 | 1.94 |
| headset_mean (set size = 8) | -7.83 | 7.94 |
| headset_mean (set size = 16) | -10.39 | 9.14 |
| headset_mean (set size = 24) | -10.30 | 8.78 |
| gaze_mean (set size = 8) | -3.70 | 4.23 |
| gaze_mean (set size = 16) | -5.41 | 5.66 |
| gaze_mean (set size = 24) | -6.86 | 7.40 |
| headset_slope | -0.46 | 0.47 |
| gaze_slope | -0.33 | 0.41 |
| accuracy_slope | -0.25 | 0.15 |
| rt_slope | -0.07 | 0.44 |

Abrreviations: RT, reaction time.

**Supplementary Figure 2 Brain injury inpatients’ subjective experiences of VR**


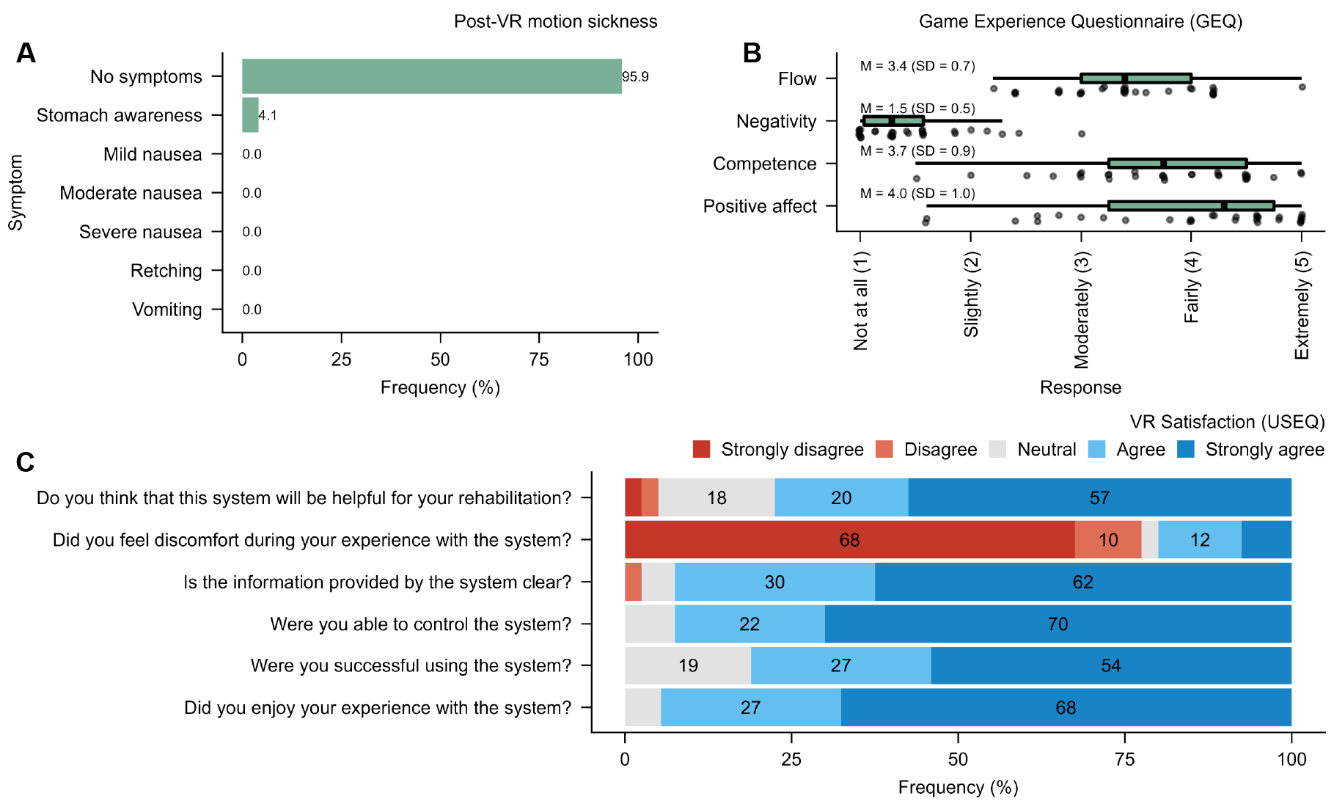


(**A**) Post-iVR motion sickness shows minimal motion sickness. (**B**) Game experience shows positive gameplay experiences with variability across patients. The bottom and top edges of each box represent the first quartile (Q1) and third quartile (Q3), respectively, indicating the interquartile range (IQR). The line within the box shows the median value. The 'whiskers' extend from the box to show the range of the data, with the lower whisker indicating the minimum value and the upper whisker indicating the maximum value. Points outside the whiskers represent outliers. (**C**) iVR satisfaction rating shows high iVR satisfaction. Please note, the analysis of this data was conducted using descriptive methods rather than inferential statistical techniques. Abbreviations: VR, virtual reality; The Game-Experience Questionnaire-Revised, GEQ; M, mean; SD, standard deviation; USEQ, The USEQ: A Short Questionnaire for Satisfaction Evaluation of Virtual Rehabilitation Systems.

**Supplementary Figure 3 Controls’ subjective experiences of VR**


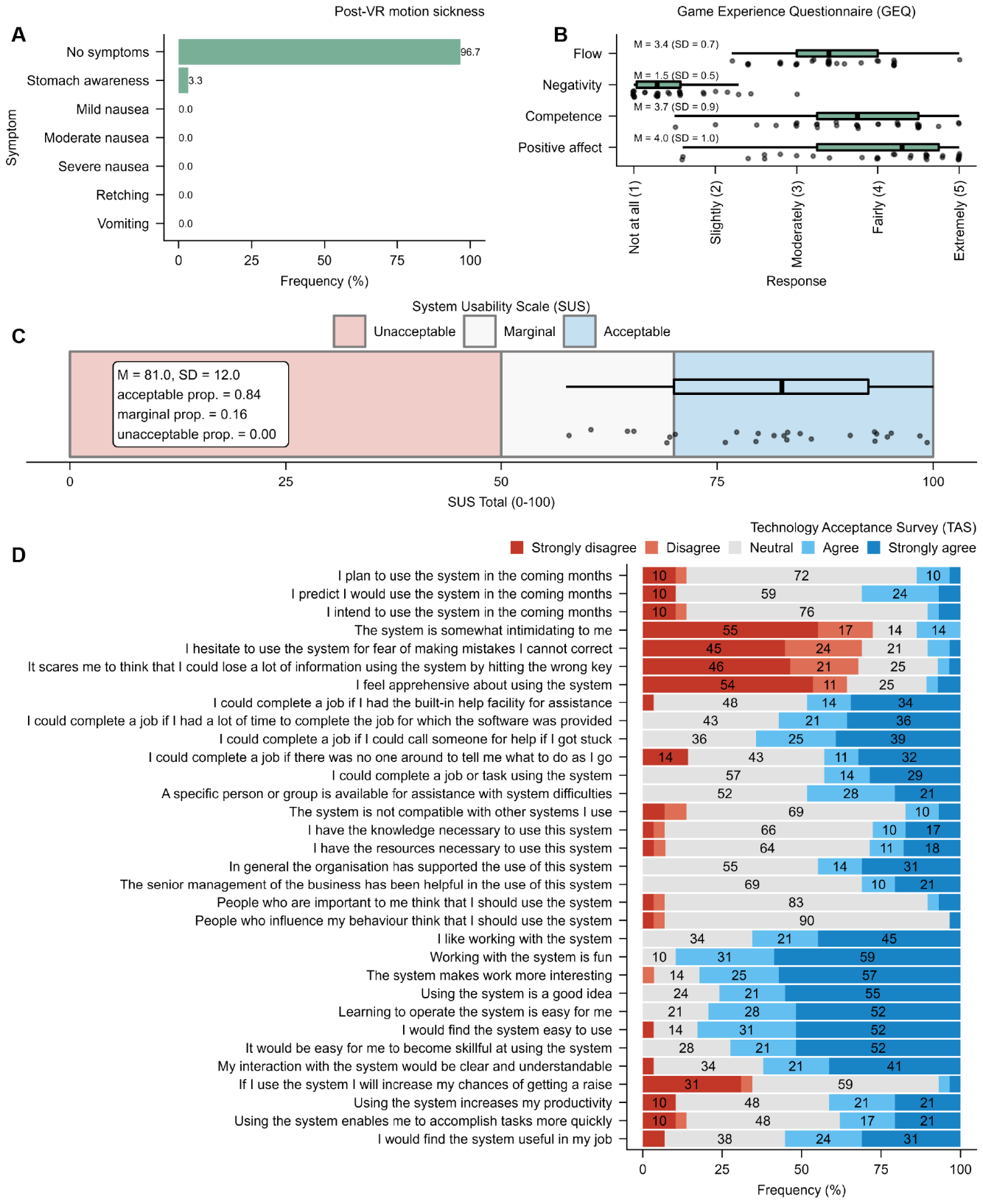


(**A**) Post-iVR motion sickness shows minimal motion sickness. (**B**) Game experience questionnaire shows overall positive game experiences with variability across controls. The bottom and top edges of each box represent the first quartile (Q1) and third quartile (Q3), respectively, indicating the interquartile range (IQR). The line within the box shows the median value. The 'whiskers' extend from the box to show the range of the data, with the lower whisker indicating the minimum value and the upper whisker indicating the maximum value. Points outside the whiskers represent outliers. (**C**) System usability shows overall acceptable system usability. (**D**) The Unified Technology Acceptance and Use of Technology scale reflects positive perceptions and clinical barriers to uptake. Please note, the analysis of this data was conducted using descriptive methods, rather than through inferential statistical techniques. The Game-Experience Questionnaire-Revised, GEQ; VR, virtual reality; M, mean; prop., proportion; SD, standard deviation; System Usability Scale, SUS; TAS, Technology Acceptance Survey (TAS).

**Supplementary Figure 4 Multidimensional not-neglect atlas maps**

**
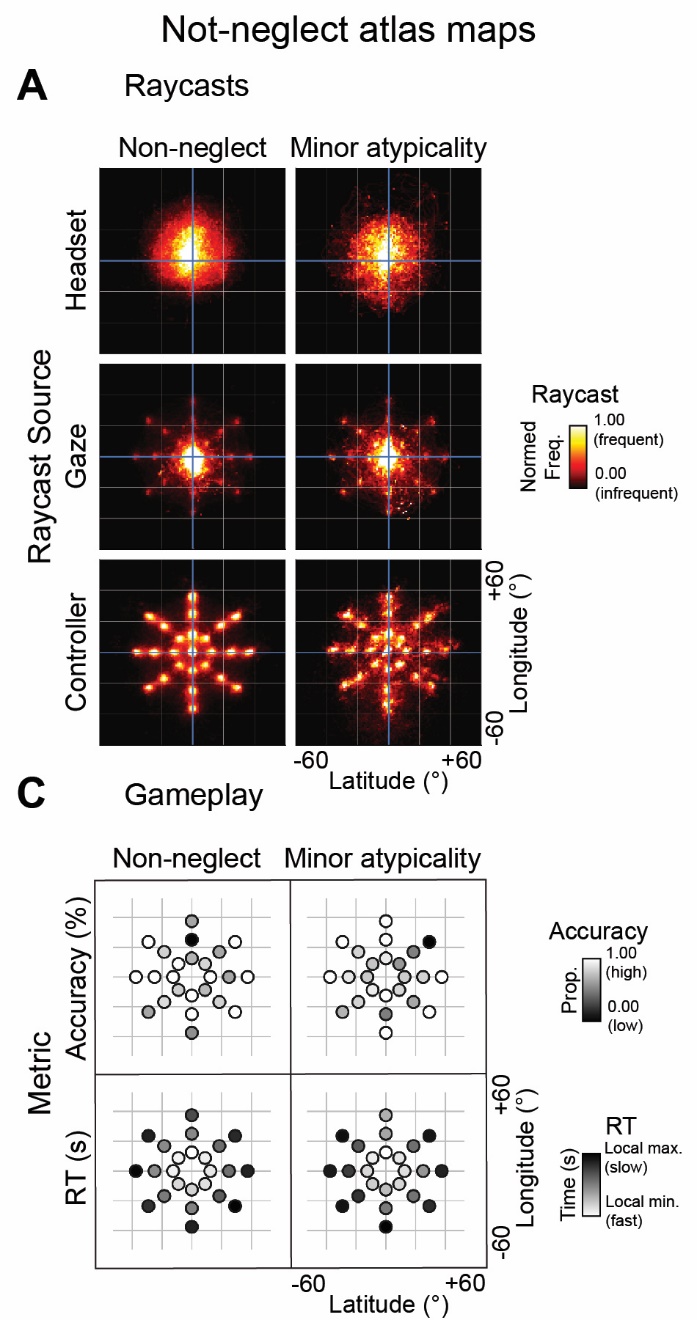
**

This figure visualises attentional patterns associated with different not-neglect patient categories, all from a first-person viewpoint. (**A**) Raycast visualisation: These maps synthesise headset, controller, and gaze data into raycast diagrams, which function as two-dimensional histograms with a 1° bin width, tailored for set size = 24 level trials. They colour-code attention frequency: high-frequency areas in whites and yellows signify areas most attended to, areas in reds indicate lesser attention, and black areas were not attended to. (**B**) Gameplay performance maps. Here, accuracy and RT metrics from gameplay are mapped by target location, each metric with its own shading scale. Lighter shades represent higher accuracy or quicker RTs (indicating better performance), while darker shades denote lower accuracy or slower RTs (poorer performance). RT data is included only for correct responses, after removing outliers. Abbreviations: Freq., frequency; L, max., maxima; min., minima; Prop., Proportion; RT, reaction time.

**Supplementary Figure 5 Multidimensional not-neglect atlas features**

**
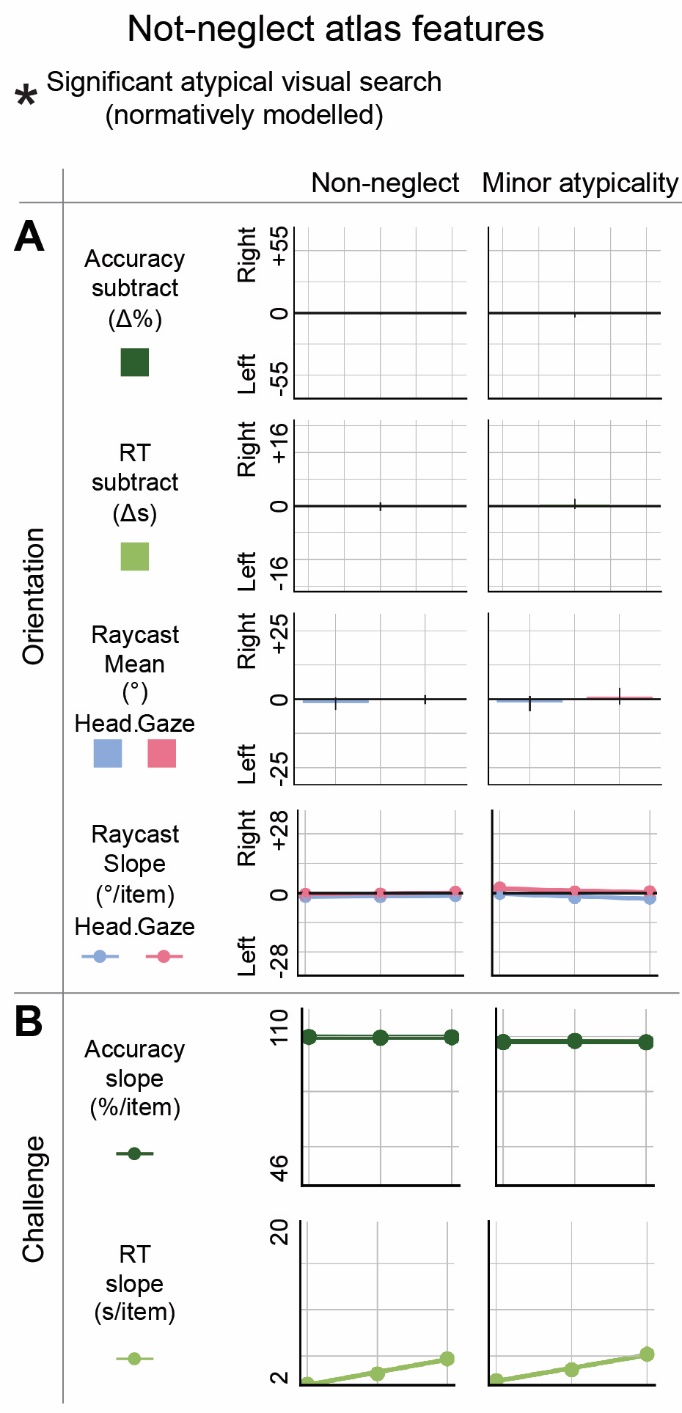
**

This figure synthesises attentional patterns into aggregate summary statistics, illustrating the basic elements of orientation and challenge responses across not-neglect patient categories. (**A**) Orientation. Demonstrated here are the orientation features, where positive values signify a rightward orientation and negative values indicate a leftward orientation. (**B**) Challenge. Results are plotted from the grand minima to grand maxima, separately for RT and accuracy. The lack of asterisks indicate the absence of atypical features among most members of each category. Atypical visual search, as identified through normative modelling, refers to gameplay that deviates from established norms for both patients and controls, with these deviations quantified as exceeding 1.5 times the interquartile range (IQR). Such cutoffs, detailed in **Supplementary Table 1,** underpin the transformation of raw scores into directional integers for orientation analyses and positive integers for evaluating challenge responses. Abbreviations: RT, reaction time; s, seconds.
